# Larvicidal activity of *Maytenus guianensis* (Celastraceae) against *Aedes aegypti* (Diptera: Culicidae)

**DOI:** 10.1590/0037-8682-0835-2020

**Published:** 2021-04-12

**Authors:** Mirilene Mendes Martins, Alyne Cunha Alves Dias, Valdir Alves Facundo, Renato Abreu Lima, Dionatas Ulises de Oliveira Meneguetti, Alexandre de Almeida e Silva

**Affiliations:** 1 Universidade Federal de Rondônia, Laboratório de Bioecologia de Insetos, Departamento de Biologia, Porto Velho, RO, Brasil.; 2 Universidade Federal de Rondônia, Programa de Pós-Graduação em Biologia Experimental, Porto Velho, RO, Brasil.; 3 Universidade Federal de Rondônia, Departamento de Química, Porto Velho, RO, Brasil.; 4 Universidade Federal do Amazonas, Departamento de Ciências, Biologia e Química, Humaitá, AM, Brasil.; 5 Universidade Federal do Acre, Colégio de Aplicação, Programa de Pós-Graduação Stricto Sensu em Ciência, Inovação e Tecnologia para a Amazônia, Rio Branco, AC, Brasil.; 6 Fundação Oswaldo Cruz Rondônia, Instituto Nacional de Epidemiologia da Amazônia Ocidental, Porto Velho, RO, Brasil.

**Keywords:** Vector control, Insecticide, Larvicidal activity, Bioprospection, 22β-hydroxytingenone, Dengue mosquito

## Abstract

**INTRODUCTION:**

Bioprospection of plant products is used to discover new insecticides.

**METHODS:**

The larvicidal activity of ethanolic extract and triterpene (tingenone B) from the bark of *Maytenus guianensis* and their effect on pupation and emergence were evaluated against *Aedes aegypti*.

**RESULTS:**

Crude extract LC_50_ was 11.3 ppm and caused ejection of the larvae intestine; tingenone B LC_50_ was 14.8 ppm. Pupation was reduced by 20% and 10%, respectively; however, the emergence was not affected.

**CONCLUSIONS:**

The crude bark extract exhibited a higher larvicidal effect against the vector.

The *Aedes aegypti* (Diptera: Culicidae) mosquito transmits several arboviruses, such as yellow fever, dengue, chikungunya, and zika. Chemical control strategies for this mosquito include natural products derived from plants as potential insecticides^1^.

Recently, the insecticidal activity of plants against mosquitoes has been immensely explored, thereby revealing that some plants display remarkable larvicidal activity against different vector mosquitoes (Pavela et al.^2^ for a review) and act as potential sources of new insecticides. The Celastraceae family is represented by four genera; among these, *Maytenus*, the largest genus with 225 species, includes *Maytenus guianensis*, an endemic species from the Amazon, popularly known as chichuá, which presents several biological activities, such as leishmanicidal and antibacterial properties^3^ ^,^ ^4^.

The present study evaluated the insecticidal effect of ethanolic extract of *M. guianensis* (Celastraceae) bark and of tingenone B, an isolated triterpene with proven biological activity^3^ against *A. aegypti,* due to their potential application in biotechnology.

Colonized *A. aegypti* were reared according to a previously described lab methodology^5^. Briefly, females of different generations were fed artificial feeders^6^ and researchers’ blood. After the blood feed, females were placed in cages and were fed 10% sucrose soaked in cotton. Dark 50 ml plastic cups were placed inside the cages, lined with filter paper to collect eggs for 3 days. The eggs were placed in 25 × 5 × 6 cm plastic trays with 1000 ml of dechlorinated water, which were cleaned every 3 days. First larval instars (L1 and L2) were fed ground TetraMin Tropical Flakes fish food. From instars L3 and L4, larvae were separated for larvicidal tests. The experiments were carried out in the laboratory at a temperature of 27-28 ºC and a 12-h photoperiod. The research was approved by the Research Ethics Committee of the Fundação Oswaldo Cruz, protocol number 73001316.4.0000.5248.

*M. guianensis* bark was collected from the Adolpho Ducke Forest Reserve, located at 26 km on the Manaus Itacoatiara road (AM-010) (latitude 02º53 'S, longitude 59º58 'W) in Manaus, Amazonas state. The species was identified by Dr. José Eduardo da Silva from the Herbarium of the National Research Institute of the Amazon (Instituto Nacional de Pesquisas da Amazônia [INPA]), and exsiccate no. 188,485 was sent to the Laboratory of Natural Products Chemistry at the Federal University of Rondônia (Universidade Federal de Rondônia [UNIR]).

Dried and ground bark was placed in a Soxhlet extractor, and hexane, chloroform, ethyl acetate, methanol, and ethanol were used to produce crude extracts. The ethanolic extract presented the highest yield and was used in the present study. Tingenone B (22β-hydroxytingenone) was obtained from the hexanic extract. We separated the hexanic extract by using silica gel column chromatography, and eluted it with n-hexane, followed by a mixture of n-hexane: CHCl_3_, which had greater polarity. Moreover, we determined the structures of all isolated compounds by analyzing their spectral data (IR, MS, ^1^H, and ^13^C, including COSY, HMQC, HMBC, and NOESY spectra) and comparing them with the existing literature data^3^.

For larvicidal tests, the crude extract was solubilized in dimethyl sulfoxide PA (1%) and tingenone B in ethanol PA (1%). Five different concentrations of the crude extract (30, 22, 18, 16, and 14 ppm) and of the isolated substance (30, 25, 20, 15, and 10 ppm) were used to calculate the lethal concentrations LC_50_ and LC_90_. Control groups for crude extract and tingenone B were dimethyl sulfoxide PA (1%) and ethanol PA (1%), respectively.

Furthermore, 25 L3/L4 instar larvae were used for each concentration during the bioassay. The larvae were transferred to 150 ml plastic cups, with 100 ml of tested solution each, and were monitored at 24-h intervals for up to 96 h of exposure to record the mortality^7^. During the follow-up period, the larvae were fed reptile food grains (Reptolife®). 

Later, 96 h after the larvicidal test, we cleaned the containers and followed the live larvae up to the pupa stage, to calculate the pupation rate by dividing the number of pupae by the number of surviving larvae. The pupae were transferred to disposable cups with 10 ml of dechlorinated water and placed in screened cages until the adults emerged. We calculated the emergence rate by dividing the number of adults by the number of pupae.

Three replicates of the bioassays were performed with four repetitions, on different days with different generations. The temperature during the experiments was 25 °C -28 °C with a 12-h photoperiod and 70%-80% humidity.

We employed the probit method to analyze the data from the mortality assays (dosage × mortality) to obtain LC_50_ and LC_90_, using the Minitab 14 (MINITAB LLC.). The effects of different concentrations of bark extract and tingenone B on larval mortality were examined via one-way analysis of variance (ANOVA); the pupation and emergence rates were analyzed with the Kruskal-Wallis test (nonparametric ANOVA), and data was compared using the Tukey’s test with Prism 8 (GraphPad LLC).

The average larval mortality rate was significantly affected by the crude bark extract (*F* = 367.3; *p* < 0.0001) and the isolated substance (tingenone B; *F* = 33.83; *p* < 0.0001) of *M. guianensis*, with a significant increase in larval mortality corresponding with increased concentration (Figure 1).

**FIGURE 1:**
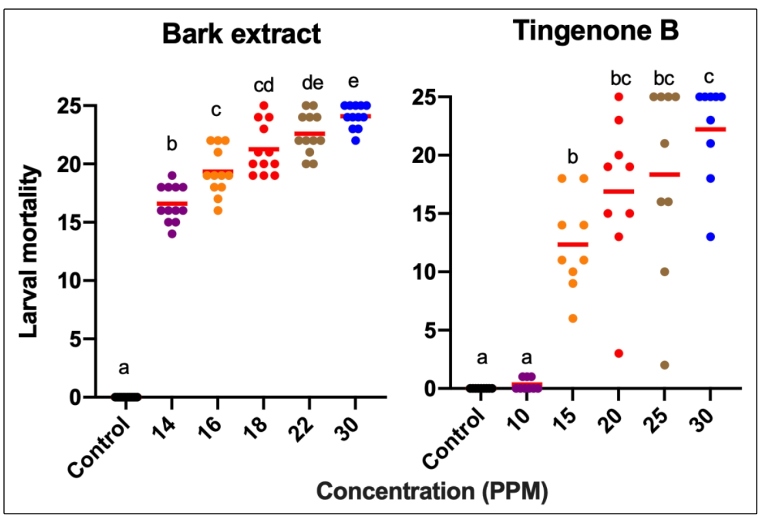
Mortality rates of *Aedes aegypti* larvae exposed to different concentrations of *Maytenus guianensis* crude bark extract and tingenone B after 48 h. Different letters indicate significant differences between concentrations (*p* < 0.05). The red lines indicate the average concentration.

The lowest concentrations of the crude extract (14 ppm) and tingenone B (10 ppm) exhibited larval mortality rates of 66% and 3%, respectively. In contrast, the highest concentration (30 ppm) of crude extract and tingenone B revealed mortality rates higher than 80%.

Interestingly, the exposure of larvae to the crude extract led to a total ejection of the digestive tract by the larvae (Figure 2).

**FIGURE 2:**
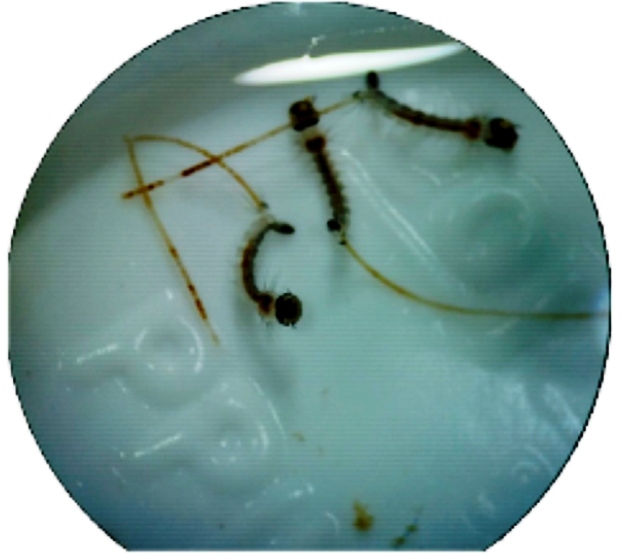
*Aedes aegypti* larvae with ejected intestine after contact with *Maytenus guianensis* crude bark extract. **Photograph:** Mirilene Martins, 2019.

Lethal concentrations (LC_50_ and LC_90_) required to kill larvae were lower for the *M. guianensis* bark extract, that is, 11.3 ppm (lower and upper CI = 10.1-12.2 ppm) and 21.4 ppm (lower and upper CI = 20.2-23.1 ppm), respectively, compared to those of tingenone B, that is, 14.8 ppm (lower and upper CI = 12.1-16.6 ppm) and 31.2 ppm (lower and upper CI = 29.4-33.8ppm), respectively.

In general, larval exposure during the experimental period significantly reduced the pupation rate (*H* = 16.9; *p* = 0.0002) by approximately 20% and 10% for crude extract and tingenone B, compared to the control; however, no significant effect was observed on adult emergence (*H* = 3.63; *p* = 0.16) (Figure 3). 

**FIGURE 3:**
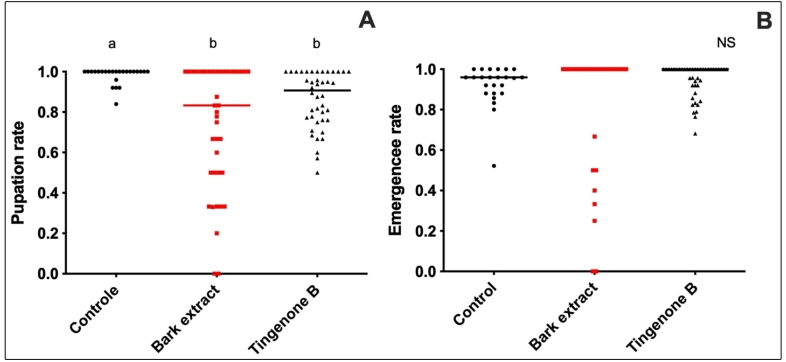
Pupation rate **(A)** and emergence rate **(B)** of *Aedes aegypti* from larvae exposed to *Maytenus guianensis* crude ethanolic bark extract and tingenone B. Different letters indicate significant differences between the groups (*p* < 0.05). NS = Not significant (*p* > 0.05). Lines indicate the median.

Although no evidence exists on the insecticidal effect of *M. guianensis* on *A. aegypti*, studies have reported that ethyl acetate extract from the stem of *Maytenus oblongata* at a concentration of 100 ppm killed 95% and 83% of the strains of Paea (susceptible to pyrethroids) and Cayenne (resistant to pyrethroids) of this species, respectively^8^. In contrast, ethanolic extract of *Maytenus rigida* leaves at 500 ppm exhibited only 15% mortality rate on the mosquito larvae^9^. Nevertheless, in our experiment, the ethanolic extract of the *M. guianensis* bark efficiently killed 96% of the larvae larvae in concentrations that were 3 and 16 times lower compared to those used in the previous studies, respectively. 

Furthermore, in tests carried out with crude ethanolic extracts from the bark of *M. guianensis*, Macari et al.^10^ reported an LC_50_ of 1230 ppm for larvae of the crustacean *Artemia franciscana* in 20 h, which was approximately 100 times greater than that observed for larvae of *A. aegypti*; however, the bark extract samples with medium- and low-polarity solvents, for example, chloroform, had remarkably lower LCs (17 ppm), suggesting that compounds, such as tingenone B, with a higher insecticidal effect, may be found in extracts with lower polarity. 

Additionally, the insecticidal effect also varies in different parts of a *Maytenus* plant, since the ethanolic extracts of *Maytenus boaria* seeds applied under mulberry leaves (*Rubus ulmifolius*) displayed an average insecticidal activity of about 80% against mulberry weevils (*Aegorhinus superciliosus*), but less than 40% when the bark extract of the same species was used^11^. Therefore, the chemical composition of different parts of the *Maytenus* plant may contribute to marked differences in its insecticidal effect, and these need to be further investigated.

To date, no studies have reported the insecticidal activity of tingenone B; however, Meneguetti et al.^3^reported that this substance was the most active triterpene of *M. guianensis* and completely inhibited the growth of *Leishmania amazonensis* promastigotes at 100 ppm concentration. Interestingly, the lepidopteran larvae *Cydia pomonella,* fed on a diet containing a similar molecule, 20-α-hydroxytingenone, presented an LC_50_ of 13.0 mg/mL (13,000 ppm) after 5 days of feeding^12^; whereas for *A. aegypti*, the LC_50_ was 15 ppm after 48 h of contact, thereby suggesting that changes in the position of functional groups are related to the insecticidal potential of these triterpenes.

Besides its larvicidal effect, exposure to the bark ethanol extract and tingenone B also reduced the *A. aegypti* pupation rate, which might be related to the deterrent (antifeeding) effect that has already been reported for extracts from other *Maytenus* and insect species^13^.

In conclusion, the *M. guianensis* bark extract had lower LC_50_ and LC_90_ values for *A. aegypti* compared to tingenone B, suggesting that other relevant insecticidal molecules, besides tingenone B, are present in the crude extract and cause morphological changes as well as potentially synergistic effects leading to the death of *A. aegypti* larvae (Figure 2). Previous studies have reported other molecules in *M. guianensis* bark extracts, including friedelin, friedenol, 16β-hydroxyfriedelin, 29-hydroxyfriedelin, tingenone, and 22β-hydroxypristimerin^14^; these should be tested alone or in combination in future experiments when available.
